# Strong Public Desire for Quality and Price Transparency in Shoulder Arthroplasty

**DOI:** 10.7759/cureus.30396

**Published:** 2022-10-17

**Authors:** Mariano E Menendez, Nicholas R Pagani, Richard N Puzzitiello, Michael A Moverman, Suleiman Y Sudah, Surena Namdari, Andrew Jawa

**Affiliations:** 1 Department of Orthopedic Surgery, Rush University Medical Center, Chicago, USA; 2 Department of Orthopedic Surgery, New England Baptist Hospital, Tufts University School of Medicine, Boston, USA; 3 Department of Orthopaedic Surgery, New England Baptist Hospital, Tufts University School of Medicine, Boston, USA; 4 Department of Orthopedic Surgery, Monmouth Medical Center, Long Branch, USA; 5 Shoulder and Elbow Surgery, Rothman Orthopaedic Institute, Philadelphia, USA; 6 Shoulder and Elbow Surgery, New England Baptist Hospital, Tufts University School of Medicine, Boston, USA

**Keywords:** amazon turk, cost, outcomes, registry, shoulder arthroplasty

## Abstract

Introduction: Concerted efforts to optimize outcomes and data transparency in shoulder arthroplasty have led to the creation of the American Academy of Orthopaedic Surgeons (AAOS) Shoulder and Elbow Registry, the first nationwide registry of its kind. We used online crowdsourcing to explore the general public’s perceptions and beliefs toward the disclosure of quality and price data in shoulder arthroplasty.

Methods: A total of 498 participants recruited using Amazon Mechanical Turk (MTurk) completed a survey regarding beliefs about public disclosure of quality and price data in shoulder arthroplasty. The MTurk is an online marketplace for crowdsourcing tasks (e.g., answering surveys) to a pool of over 500,000 registered workers in exchange for financial compensation. Requesters post human-intelligence tasks, and workers can respond to those that they are interested in completing. This web-based platform is an efficient survey tool for medical research, with comparable national representativeness to traditional convenience samples.

Results: The majority (95%) of respondents believed surgeons and hospitals should share their data with national registries such as the AAOS Shoulder and Elbow Registry. Most believed that patients considering shoulder arthroplasty should have public access to surgeons’ outcomes and complication rates (96%), years of experience (95%), and case volume (92%). Most respondents desired price transparency in implant costs (95%), surgeon reimbursement (80%), and hospital reimbursement (84%). In decreasing order of importance, the top three factors guiding surgeon choice were: (1) surgeon included in the insurer’s network, (2) annual case volume, and (3) publicly available outcomes.

Conclusion: Increased quality and price transparency in shoulder arthroplasty may empower patients to make better-informed decisions about their care and ultimately enhance value. Given the strong public desire for data transparency and the notion that public disclosure of data is intrinsically associated with performance improvement, surgeons and hospitals should strongly consider submitting their data to national registries such as the AAOS Shoulder and Elbow Registry.

## Introduction

Optimizing health outcomes and the care experience after discretionary orthopaedic surgery has become a major priority in the shift toward value-based care [1,2]. Shoulder arthroplasty is an increasingly popular and costly procedure that has been catching the attention of value-based bundled payment initiatives [3-6]. Despite the growing realization that public disclosure of data can maximize value [7], patients seeking shoulder surgery have very limited information about the quality and cost of care they provide. This contrasts with other industries such as consumer goods (e.g., electronics, home appliances), where customers can use extensive quality and price data to make informed decisions.

Recent efforts spearheaded by the American Academy of Orthopaedic Surgeons (AAOS) to enhance outcomes and data transparency have led to the creation of the Shoulder and Elbow Registry, the first nationwide registry of its kind. Conceived in 2018, the AAOS Shoulder and Elbow Registry collects procedural and patient-specific data (including patient-reported outcome measures) for shoulder arthroplasty, elbow arthroplasty, and rotator cuff repair procedures [8]. Based on the 2019 Annual Report [9], there were more than 85 participating facilities spanning 20 states across the United States -accounting for more than 7,800 patient procedures - and this number has since continued to rise. While the concept of increased public transparency of quality and price data holds promise in empowering patients to make more informed, value-driven decisions, the extent to which the general public is interested in this information and finds it useful is unclear.

This study sought to explore the United States general public’s perceptions and beliefs toward disclosure of quality and price data in shoulder arthroplasty.

## Materials and methods

Survey design

We conducted an online cross-sectional survey to explore the United States general public’s perceptions and beliefs toward the disclosure of quality and price data in shoulder arthroplasty. The survey was developed using Qualtrics (Provo, UT) and consisted of 21 questions designed to be completed in less than 10 minutes (Supplementary Appendix).

We collected basic demographic data including age, sex, race/ethnicity, primary language, education level, marital status, annual income, insurance status, and region of residence. We also gathered data about perceived overall health status, health literacy (using the Single Health Literacy Screening Question) [10], and patient engagement (using the Single Health Confidence Question) [11]. Limited health literacy was defined as answers of "somewhat/a little/not at all" to the Single Health Literacy Screening Question "How confident are you filling out medical forms by yourself?" [12]. Low patient engagement was defined as an answer of "not very confident" to the Single Health Confidence Question "How confident are you that you can control and manage most of your health problems?" [11].

All the questions used to assess perceptions and beliefs towards public disclosure of shoulder arthroplasty data were based on a four-point Likert scale ("strongly disagree/disagree/agree/strongly agree") to optimize symmetry and balance. This scale was chosen as an effective means of assigning a numeric value to the opinions of a sample population. An iterative consensus process was used to formulate them. The answers "agree" and "strongly agree" were considered together when reporting general result trends. These questions asked respondents whether surgeons and hospitals offering shoulder arthroplasty should share their data with national registries, and whether patients considering shoulder arthroplasty should have public access to surgeons’ outcomes, complications, years of experience, annual case volume, and surgery-related costs (Table 1). In this study, the quality of shoulder arthroplasty is reflected in the study participants' desire to have a surgeon's annual case volume, years of experience, complications, and surgical outcomes publicly available.

**Table 1:** Survey participant characteristics (n=498) ^†^Defined as answers of "somewhat/a little/not at all" to the Single Health Literacy Screening Question. ^⨏^Defined as an answer of "not very confident" to the Single Health Confidence Question.

Variable, n (%)	Respondents
Age in years	
<25	72 (14.5)
25–40	308 (61.8)
41–60	99 (19.9)
>60	19 (3.8)
Sex	
Female	243 (48.8)
Male	355 (51.2)
Race	
White	345 (69.3)
Black	51 (10.2)
Hispanic	31 (6.2)
Asian	63 (12.7)
Other	8 (1.6)
Native English speaker	
Yes	474 (95.2)
No	24 (4.8)
Marital status	
Single	179 (35.9)
Separated/divorced	26 (5.2)
Married	290 (58.2)
Widowed	3 (0.6)
Annual income in US$	
<30,000	151 (30.3)
30,000-60,000	205 (41.2)
>60,000	142 (28.5)
United States region	
Northeast	141 (28.3)
Midwest	84 (16.9)
South	190 (38.2)
West	83 (16.7)
Highest education level	
Less than high school	1 (0.2)
High school	109 (21.9)
College degree	219 (44.0)
Graduate degree	169 (33.9)
Primary health insurance	
Medicare	151 (30.3)
Medicaid	78 (15.7)
Private	237 (47.6)
Veterans’ affairs	7 (1.4)
Uninsured	25 (5.0)
Perceived overall health status	
Poor or fair	32 (6.4)
Good	227 (45.6)
Very good or excellent	239 (48.0)
Limited health literacy^†^	126 (25.3)
Low patient engagement^⨏^	38 (7.6)

The last question of the survey asked respondents to choose the main factor guiding surgeon choice among six options: (1) online patient reviews, (2) publicly available outcomes, (3) bedside manner, (4) fellowship training, (5) annual case volume, and (6) surgeons included in the insurer’s network.

Participant enrollment

Participants were recruited using Amazon Mechanical Turk (MTurk; Amazon.com, Inc., Seattle, WA), an online crowd-sourcing marketplace that allows people to complete human intelligence tasks (e.g., answering surveys, extracting text from documents, moderate content) for small amounts of money [13]. With a pool of over 500,000 registered users, this web-based platform has been increasingly used for medical research given its high efficiency, relative inexpensiveness, and comparable national representativeness to traditional convenience samples [14,15].

Our survey was limited to adults (≥18 years) with valid social security numbers currently residing in the United States. Each participant was awarded US$ 0.12 for completing our survey. To maximize demographic representation and therefore generalizability of the survey results, we targeted enrollment of 550 participants. At the end of the survey, we included a randomly generated unique completion code as an attention check and to ensure that respondents were humans and not bots. Respondents were not included in our study if the typed code was different from that generated by our algorithm. The survey was administered over a two-day period in May 2020. This study was exempt from review by our Institutional Review Board.

## Results

The responses of 498 participants were considered in our study (Table 1). Most respondents (76%) were under the age of 40, and 51% were male. Sixty-nine percent were white, followed by Asian (13%), black (10%), and Hispanic (6%) respondents. Overall, they were well-educated, with 78% reporting at least a college degree. The majority (95%) spoke English as their primary language and were either single (36%) or married (58%). The South was the region with the highest number of responses (38%), followed by the Northeast (28%), the Midwest (17%), and the West (17%). Nearly half (48%) considered themselves in very good or excellent health. One in four (25%) exhibited limited health literacy based on the Single Health Literacy Screening Question, and 7.6% showed low engagement in their care according to the Single Health Confidence Question.

The majority (95%) of respondents believed surgeons and hospitals should share their data with national registries such as the AAOS Shoulder and Elbow Registry (Table 2). Most believed that patients considering shoulder arthroplasty should have public access to surgeons’ outcomes and complication rates (96%), years of experience (95%), and case volume (92%). Most respondents desired price transparency in implant costs (95%), surgeon reimbursement (80%), and hospital reimbursement (84%).

**Table 2:** Public perceptions of disclosure of quality and price data in shoulder arthroplasty (n=498)

S. No.	Question/statement	Respondents, n (%)			
		Strongly disagree	Disagree	Agree	Strongly agree
(1)	Surgeons and hospitals offering shoulder replacement surgery should share their data with national registries	5 (1.0)	19 (3.8)	309 (62.0)	165 (33.1)
(2)	Patients considering shoulder replacement should have public access to surgeons’ outcomes and complication rates	3 (0.6)	17 (3.4)	241 (48.4)	237 (47.6)
(3)	Patients considering shoulder replacement should have public access to surgeons’ years of experience	1 (0.2)	23 (4.6)	240 (48.2)	234 (47.0)
(4)	Patients considering shoulder replacement should have public access to surgeons’ annual case volume	5 (1.0)	37 (7.4)	250 (50.2)	206 (41.4)
(5)	Patients considering shoulder replacement should have public access to total surgery-related costs	5 (1.0)	28 (5.6)	192 (38.6)	273 (54.8)
(6)	Patients considering shoulder replacement should have public access to implant costs	5 (1.0)	19 (3.8)	207 (41.6)	267 (53.6)
(7)	Patients considering shoulder replacement should have public access to surgeon fees	14 (2.8)	84 (16.9)	253 (50.8)	147 (29.5)
(8)	Patients considering shoulder replacement should have public access to hospital fees	10 (2.0)	69 (13.9)	241 (48.4)	178 (35.7)

**Figure 1:**
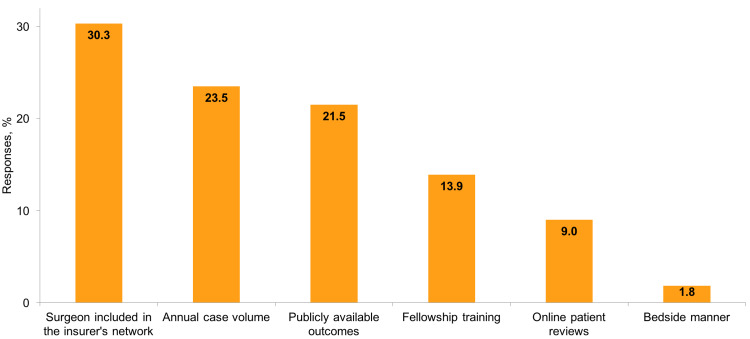
What is the main factor guiding your choice of an arthroplasty surgeon?

In decreasing order of importance, the top 3 factors guiding surgeon choice were: (1) surgeon included in the insurer’s network, (2) annual case volume, and (3) publicly available outcomes (Figure 1). The factor with the lowest impact on surgeon choice was bedside manner.

## Discussion

Patients considering elective orthopaedic surgery are expected to make complex health decisions with limited information available to them. Recent nationwide efforts to optimize outcomes and standardize data collection in shoulder arthroplasty have led to the creation of the AAOS Shoulder and Elbow Registry in 2018. With the transition of high-quality data to actionable knowledge, ensuring patient access to this information has the potential to maximize value in shoulder arthroplasty. But it is unclear the degree to which the public finds this information important and useful when making healthcare decisions. In this context, we used online crowdsourcing to report large-scale public perceptions and beliefs toward the disclosure of quality and price data in shoulder arthroplasty.

The principal strengths of our study include the national representativeness provided by MTurk. This crowdsourcing platform is a powerful and relatively inexpensive way of obtaining survey results that have arguably closer generalizability to the US population than those derived from traditional survey methods [15,16]. Nonetheless, our analysis was subject to several shortcomings. First, it is known that MTurk users tend to be more technologically savvy, educated, and slightly younger compared to the general population, which might have perhaps biased their responses towards desiring more information to make health decisions [17]. While it is possible that our study findings might have been somewhat different from those of another study population, one would not expect major discrepancies in the results given that the distribution of responses was largely maintained across different demographic subgroups. Second, there was no calculable response rate as we were blinded to the overall number of viewers of the survey who chose not to participate. Furthermore, we do not know whether these individuals differed demographically from those who elected to participate. Finally, this survey relied on the respondents’ ability to imagine their responses to hypothetical scenarios rather than evaluating the answers of real patients facing these diagnoses and decisions [13].

Our findings highlight the strong public desire for quality and price transparency in shoulder arthroplasty. The vast majority of respondents felt that providers should share their data with national registries, and believed that patients seeking shoulder surgeons should have access to their outcomes, complications, years of experience, annual case volume, and procedure-related fees. It is interesting that there are very little data available to patients considering shoulder arthroplasty, despite the notion that informed patients make more value-driven decisions. This information void suggests ample opportunity for optimizing value in shoulder arthroplasty. Recent efforts to collect high-quality data, such as the AAOS Shoulder and Elbow Registry, have the potential to improve patient engagement and reduce decisional conflict. In addition to the Shoulder and Elbow Registry, the AAOS houses three other registries: the American Joint Replacement Registry, the Musculoskeletal Tumor Registry, and the American Spine Registry [18,19]. Public disclosure of these data might enable patients to choose high-quality hospitals and surgeons and to be more involved in their decisions.

There is also growing evidence that publicly reporting performance motivates clinicians to improve performance [20,21]. For instance, Bozic et al. [22] used Medicare data to show a 33% decrease in complication rates and a 25% reduction in readmission rates after total joint arthroplasty during a period coinciding with the start of public reporting of these measures. It is possible that public reporting triggers providers to pursue improvement, by raising awareness of previously unrecognized quality deficits or to protect or improve their reputation and patient acquisition [23]. However, one important point that merits consideration is the need for robust risk-adjustment methods when reporting provider-specific outcomes that could influence patient decisions and payment models [24,25]. Without appropriate risk adjustment, public reporting might have the unintended consequence of encouraging cherry-picking of more profitable, lower-risk patients [26].

We observed that the biggest factor guiding surgeon choice was in-network coverage, which is not surprising given that out-of-network bills can be very large and extremely impactful for those who receive them. A recent study by Chhabra et al. [27] raises awareness of the important concept of "surprise" billing in elective surgery, in which patients receiving care from in-network surgeons and hospitals receive unexpected bills from other out-of-network clinicians (e.g., anesthesiologists, surgical assistants) they did not choose. In their analysis, approximately one in five patients undergoing common elective operations received an unexpected out-of-network bill, with a mean financial liability of more than $2,000 [27]. Given how this could negatively affect the patient experience, surgeons should ensure that all the personnel involved in the care team accept the same insurance plans, and Congress should work toward eliminating surprise billing [28]. The other two most important data points influencing surgeon choice were annual case volume and publicly available outcomes, both of which are currently unavailable to patients seeking shoulder surgeons. While patients are increasingly expected to take a more active role in health decisions, the data available to them are scarce. Our study raises awareness of the important role that public disclosure of data collected in national registries such as the Shoulder and Elbow Registry might have in enhancing patient engagement and outcomes.

There are several limitations to this study. First, as the majority of study participants were young, healthy, and college educated, the risk of selection bias was high. The generalizability of our study could be improved by including patients who had previously undergone shoulder arthroplasty. Second, while our survey data indicates that insurance type is the most heavily weighed factor in choosing a shoulder arthroplasty surgeon, this claim is not based on surgeon data or outcomes. In addition, despite the assessment of public desire for price and quality information for shoulder arthroplasty, our survey did not include specifics pertaining to cost or national registry data.

## Conclusions

This study used online crowdsourcing to underscore the strong public desire for health data transparency and choice. Increasing quality and price transparency in shoulder arthroplasty may empower patients to make better-informed decisions about their care and ultimately enhance value. Given the strong interest in data transparency and the notion that public reporting of data is intrinsically associated with performance improvement, surgeons and hospitals should strongly consider submitting their data to national registries such as the AAOS Shoulder and Elbow Registry.

## Appendices

**Table 3:** ASES Registry Survey

Item No.	Question	Strongly disagree	Disagree	Agree	Strongly agree
1	Surgeons and hospitals offering shoulder replacement surgery should share their data with national registries				
2	Patients considering shoulder replacement should have public access to surgeons’ outcomes and complication rates				
3	Patients considering shoulder replacement should have public access to surgeons’ years of experience				
4	Patients considering shoulder replacement should have public access to surgeons’ annual case volume				
5	Patients considering shoulder replacement should have public access to total surgery-related costs				
6	Patients considering shoulder replacement should have public access to implant costs				
7	Patients considering shoulder replacement should have public access to surgeon fees				
8	Patients considering shoulder replacement should have public access to hospital fees				

Demographic Information of ASES Registry Survey Participants

1. Age

o   <25

o   25-40

o   41-60

o   >60

2. Sex

o  Male

o  Female

3. Is English your first language?

o  Yes

o  No

4. Marital Status

o   Single

o   Married

o   Seperated/divorced

o   Widowed

5. Highest educational degree

o   Did not finish high school

o   High school degree

o   College degree

o   Graduate degree

6. Annual income

o   <30k

o   30-59k

o   >60k

7. Region of residence in the US

o   Northeast

o   Midwest

o   South

o   West

8. Race/ethnicity

o   White

o   Black

o   Hispanic

o   Asian

o   Other

9. How confident are you filling out medical forms by yourself?

o   Not at all

o   A little

o   Somewhat

o   Quite a bit

o   Extremely

10. How confident are you that you can control and manage most of your health problems?

o   Not very confident

o   Somewhat confident

o   Very confident

o   I do not have any health problems

11. Health Insurance

o   Private or commercial

o   Medicare

o   Medicaid

o   Military or Veterans Administration

o   None

12. How is your overall health status?

o   Poor or Fair

o   Good

o   Very Good

o   Excellent
